# The impact of urbanization on body size of Barn Swallows *Hirundo rustica gutturalis*

**DOI:** 10.1002/ece3.7088

**Published:** 2020-12-19

**Authors:** Yanyan Zhao, Yu Liu, Elizabeth S. C. Scordato, Myung‐Bok Lee, Xiaoying Xing, Xinyuan Pan, Yang Liu, Rebecca J. Safran, Emilio Pagani‐Núñez

**Affiliations:** ^1^ State Key Laboratory of Biocontrol School of Life Sciences/School of Ecology Sun Yat‐sen University Guangzhou China; ^2^ College of Life Sciences Beijing Normal University Beijing China; ^3^ Department of Ecology and Evolutionary Biology University of Colorado Boulder CP USA; ^4^ Biological Sciences Department California State Polytechnic University Pomona CA USA; ^5^ Guangdong Institute of Applied Biological Resources Guangzhou China; ^6^ College of Wildlife and Protected Area Northeast Forestry University Harbin, Heilongjiang China; ^7^ Department of Health and Environmental Sciences Xi’an Jiaotong‐Liverpool University Suzhou China

**Keywords:** body size, China, latitude, longitude, sex differences, urbanization

## Abstract

Urbanization implies a dramatic impact on ecosystems, which may lead to drastic phenotypic differences between urban and nonurban individuals. For instance, urbanization is associated with increased metabolic costs, which may constrain body size, but urbanization also leads to habitat fragmentation, which may favor increases in body mass when for instance it correlates with dispersal capacity. However, this apparent contradiction has rarely been studied. This is particularly evident in China where the urbanization process is currently occurring at an unprecedented scale. Moreover, no study has addressed this issue across large geographical areas encompassing locations in different climates. In this regard, Barn Swallows (*Hirundo rustica*) are a suitable model to study the impact of urbanization on wild animals because they are a widely distributed species tightly associated with humans. Here, we collected body mass and wing length data for 359 breeding individuals of Barn Swallow (*H. r. gutturalis*) from 128 sites showing different levels of urbanization around the whole China. Using a set of linear mixed‐effects models, we assessed how urbanization and geography influenced body size measured using body mass, wing length, and their regression residuals. Interestingly, we found that the impact of urbanization was sex‐dependent, negatively affecting males’ body mass, its regression residuals, and females’ wing length. We also found that northern and western individuals were larger, regarding both body mass and wing length, than southern and eastern individuals. Females were heavier than males, yet males had slightly longer wings than females. Overall, our results showed that body mass of males was particularly sensitive trait to urbanization, latitude, and longitude, while it only showed a weak response to latitude in females. Conversely, while wing length showed a similar geographical pattern, it was only affected by urbanization in the case of females. Further research is needed to determine whether these phenotypic differences are associated with negative effects of urbanization or potential selective advantages.

## Graphical abstract

Urbanization has become one of the defining questions of the Anthropocene. In China, our results showed that body size of Barn Swallows decreased towards the South and West and with increasing urbanization. This effect was, however, sex‐dependent, affecting wing‐length in females and body mass and body size index in males.

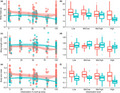

## 1 INTRODUCTION

Urbanization drives a dramatic change in environmental conditions, eliciting a broad variety of phenotypic and genetic responses by living organisms (Alberti, 2015; Johnson & Munshi‐South, 2017). Among these responses, body size variation is particularly important. Recent research using a relatively large number of ectotherm arthropod taxa has shown that, according to Atkinson's temperature‐size rule (Atkinson, 1994), urbanization drives an overall reduction in body size for most species (Merckx et al., 2018). This change was attributed to the urban heat‐island effect and to a decrease in available resources. Yet, different species showed divergent patterns, with some species decreasing and others increasing in body size. This variability in species responses may be linked to life‐history traits. Species showing high dispersal capacity and large body size are able to cope with the negative effects of urbanization and, thus, can maintain or increase their body size in urbanized habitats (Merckx et al., 2018; Santini et al., 2019; but see Evans et al., 2011, Sol et al., 2014). This suggests that different factors linked to species’ ecology and evolutionary history may result in divergent patterns of body size change across urbanization gradients. However, evidence from these processes is still scarce and more research needs to be done in order to understand how urbanization influences body size variation in wild organisms.

In animals, changes in body size at contemporary scales are commonly linked to biotic interactions, biogeographical constraints, and to changes in habitat structure (Allen et al., 2006). Additionally, Bergmann's rule predicts a negative relationship between body size and temperature, which is often manifested as a latitudinal pattern (Ashton, 2002). Urbanization, which drives a drastic transformation in environmental conditions—usually resulting in decreased food availability and increased temperature and habitat patchiness—may constrain body size. In birds, it has been shown that urbanization negatively impacts body size, nestling development, and condition measurements (e.g., Heiss et al., 2009; Herrera‐Dueñas et al., 2017; Jiménez‐Peñuela et al., 2019; Liker et al., 2008; Ruiz et al., 2002). Most works regard short‐term variation in body size as mostly dependent on resource availability and, therefore, interpreted it as phenotypic plasticity (Hendry et al., 2008; Lima, 1986; Pollock et al., 2017; Seress et al., 2020). Liker et al. (2008), however, showed in a common garden experiment that this difference probably was the result of adaptive divergence. Other studies have shown no evidence of such impact (Bókony et al., 2012; Chamberlain et al., 2009; see also Giraudeau et al., 2014, Salmón et al., 2018). Moreover, previous studies have mostly assessed this question at relatively small spatial scales. Thus, there is a need for more studies analyzing the effects of urbanization on body size using a comparative framework across different populations and broader spatial scales, incorporating a biogeographical perspective.

Barn Swallows *Hirundo rustica* are an ideal model organism to study urbanization. They are well adapted to human disturbance, similar to other human commensals, such as the House Sparrow *Passer domesticus* (Riyahi et al., 2013) and the Tree Sparrow *Passer montanus* (Zhang et al., 2011). They inhabit both urban and rural areas and are widely distributed around the world. In China, Barn Swallows are found in both temperate and tropical environments, making them particularly suitable to examine the biogeographical component of body size variation. There are two main subspecies, *H. r. rustica,* in the extremely northwest China (Xinjiang and NW Gansu province), and *H. r. gutturalis,* widely distributed in the east China (Dor et al., 2010; Liu et al., 2020; Scordato & Safran, 2014). Moreover, there is significant variation in urbanization rates across the country (Lin et al., 2015), so that individuals in developed regions may experience a stronger influence of urbanization than individuals in more remote areas (e.g., East China's urbanized coast vs. West China's sparsely populated areas). Finally, male and female Barn Swallows may show contrasting patterns of body size variation due to sex‐differential responses to urbanization. In this species, sexual dimorphism is apparent, with males usually showing smaller body size than females, and sexual selection operates with more intensity on the former (Liu et al., 2018; Safran et al., 2016).

Here, after controlling for the effect of geography, we assessed the influence of urbanization on body size variation in the subspecies *gutturalis* of Barn Swallow in China. We used several traits that may potentially be affected by urbanization to assess body size variation (see, e.g., Caizergues et al., 2018; Saccavino et al., 2018): body mass, wing length, and their regression residuals (hereinafter body size index). First, due to food and habitat constraints (Pollock et al., 2017; Seress et al., 2020) and to the heat‐island effect (Andrew et al., 2018; Scheffers et al., 2016), we predicted that urbanization would have a negative influence in body size. Second, in line with Bergmann's rule and due to the potential negative effect of high temperature on nestling development (Andrew et al., 2018; Ashton, 2002), we predicted a positive relationship between latitude and body size. These patterns, namely a decrease in body size toward highly urbanized and hot areas, could also be promoted by geographical variation in the urban heat‐island effect, which in China is stronger in southern than in northern cities (Zhou et al., 2004, 2016), and by the relatively high degree of urbanization of East China compared to West China. Finally, given that males experience stronger sexual selection and thus may have higher energetic demands than females, the negative effect of urbanization on body size could be more intense in males than in females.

## 2 METHODS

### 2.1 Study area

**Figure 1:**
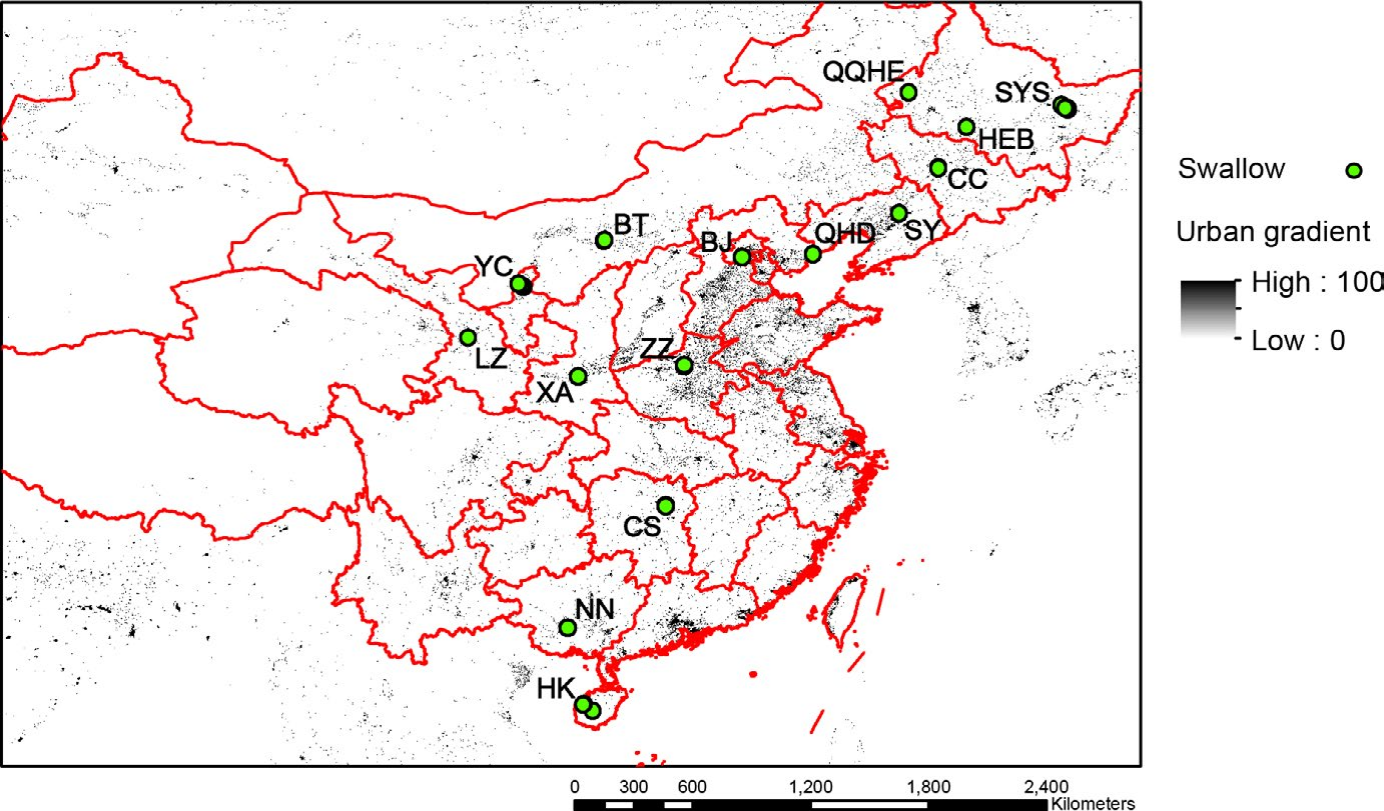
Map showing the sampling sites across 13 provinces of China (from North to South and East to West: Heilongjiang, Jilin, Liaoning, Hebei, Beijing, Henan, Inner Mongolia, Shaanxi, Ningxia, Gansu, Hunan, Guangxi and Hainan). All the sample points are clustered into 15 cities (SYS: Shuangyashan; QQHE: Qiqihar; HEB: Harbin; CC: Changchun; SY: Shenyang; QHD: Qinhuangdao; BJ: Beijing; ZZ: Zhengzhou; BT: Baotou; XA: Xi'an; YC: Yinchuan; LZ: Lanzhou; CS: Changsha; NN: Nanning; HK: Haikou)

We selected 128 sites within 13 provinces of China across a broad geographical and urbanization gradient and in different climatic regions—from subtropical in the south to humid continental in the east and dry continental in the west (Domrös & Peng, 2012). All the sites were clustered around 15 main urban areas, hereafter simply labeled as cities (SYS: Shuangyashan; QQHE: Qiqihar; HEB: Harbin; CC: Changchun; SY: Shenyang; QHD: Qinhaungdao; BJ: Beijing; BT: Baotou; YC: Yinchuan; LZ: Lanzhou; XA: Xi'an; ZZ: Zhengzhou; CS: Changsha; NN: Nanning; HK: Haikou) (Figure 1). The built‐up area within a 1‐km pixel grid around the nest was extracted for each individual bird using ArcGIS 10.1 from the dataset of Global 1‐km Consensus Land Cover (http://www.earthenv.org/) (Tuanmu & Jetz, 2014).

### 2.2 Data collection

We captured adult Barn Swallows during the breeding season from April to June 2014 and May to June 2015 by installing mist nets close to their nests after the first‐brood nestlings hatched, and banded them in order to avoid duplicates. We recorded body mass of 359 breeding adult individuals using a digital scale to the nearest 0.01 g (Pesola) and maximum‐chord wing length using a metal ruler with an end stop to the nearest mm, including 184 males and 175 females of *H. r. gutturalis*. See Liu et al. (2020) for more details on the field procedure and Table A1 for detailed information on sample size. A single person (E.S.C.S.) collected all data. We also collected a small amount of data on *H. r. rustica* and *rustica—gutturalis* hybrids, which were excluded from this study to avoid the influence of subspecies differences on body size (Liu et al., 2020).

### 2.3 Statistical analysis

We applied Moran's *I* test to assess the degree of spatial autocorrelation among our study sites. We found that these sites were not randomly distributed across our study area (Moran's *I* = 0.23, *p* < .001), so that we included study area (“city”) as random factor in all models. Furthermore, since urbanization can be rather heterogeneous across time and space, showing complex relationships with habitat features, species richness, and species’ traits (e.g., McKinney, 2008; Szulkin et al., 2020), we constructed two sets of models with built‐up area data as a continuous variable and as a categorical factor. Based on data distribution and sample size, we classified these 128 sites into four levels of urbanization. We did this according to the proportion of built‐up areas in the 1‐square‐kilometer area where they were breeding (L: low, 0%–20%, 43 sites, *N* = 94; ML: mid‐low, 21%–40%, 17 sites, *N* = 29; MH: mid‐high, 41%–60%, 35 sites, *N* = 79; H: high, 61%–100%, 33 sites, *N* = 157). This is a common and effective method to evaluate urbanized level in the study on the impact of urbanization (Newbold et al., 2015; Sol et al., 2020).

We used a linear mixed‐effect model fit by restricted maximum likelihood to assess the impact of urbanization on body size of Barn Swallows, controlling for geography, date, and sex differences. We ran three sets of models using body mass (g) (including and excluding wing length), wing length (mm), and the body size index as dependent variables. We log‐transformed body mass and wing length to approximate normality. We included latitude, longitude, and sampling date (*N* days from April 1st), which were scale‐transformed to operate with comparable values, as explanatory variables. We also included sex (male or female) as categorical factor in the full models including both sexes. We carried out a Levene's test to assess the homogeneity of variance assumption and found that sex did not violate the homoscedasticity assumption. We estimated *p*‐values using the normal approximation given the relatively large sample size of our sample (Barr et al., 2013).

Furthermore, as previously stated, we ran these three sets of models alternatively including urbanization as a continuous variable and as a categorical factor. In the latter, we included four categories of urbanization (low, mid‐low, mid‐high, and high; see previous section) as a fixed effect. We used the different levels as reference in a sequence of models to assess all the potential combinations. The effects for the rest of explanatory variables remained the same, and we display the results for all the combinations of urbanization levels in the tables. Finally, given that sampling was conducted across 2 years, year was included as random factor. We considered including climatic variables—average annual temperature and precipitation from 2011 to 2015 as explanatory variables, which were obtained from Loess plateau science data center, National Earth System Science Data Sharing Infrastructure, National Science & Technology Infrastructure of China (http://loess.geodata.cn) (Peng et al., 2019). However, we finally excluded these climatic variables due to their high correlation with latitude and longitude (Table A2).

For each dependent variable, and because we were interested in assessing differential responses to urbanization between the sexes without overloading the models with an excessive number of interaction factors, we ran a full model and then one separate model for each sex.

All analyses were carried out in R 4.0.2 (R Core Team, 2020) using the packages spdep 1.1‐5 (Bivand & Wong, 2018), lme4 1.1‐21 (Bates et al., 2015), and car 3.0‐4 (Fox & Weisberg, 2018).

## 3 RESULTS

### 3.1 Body mass

**Figure 2:**
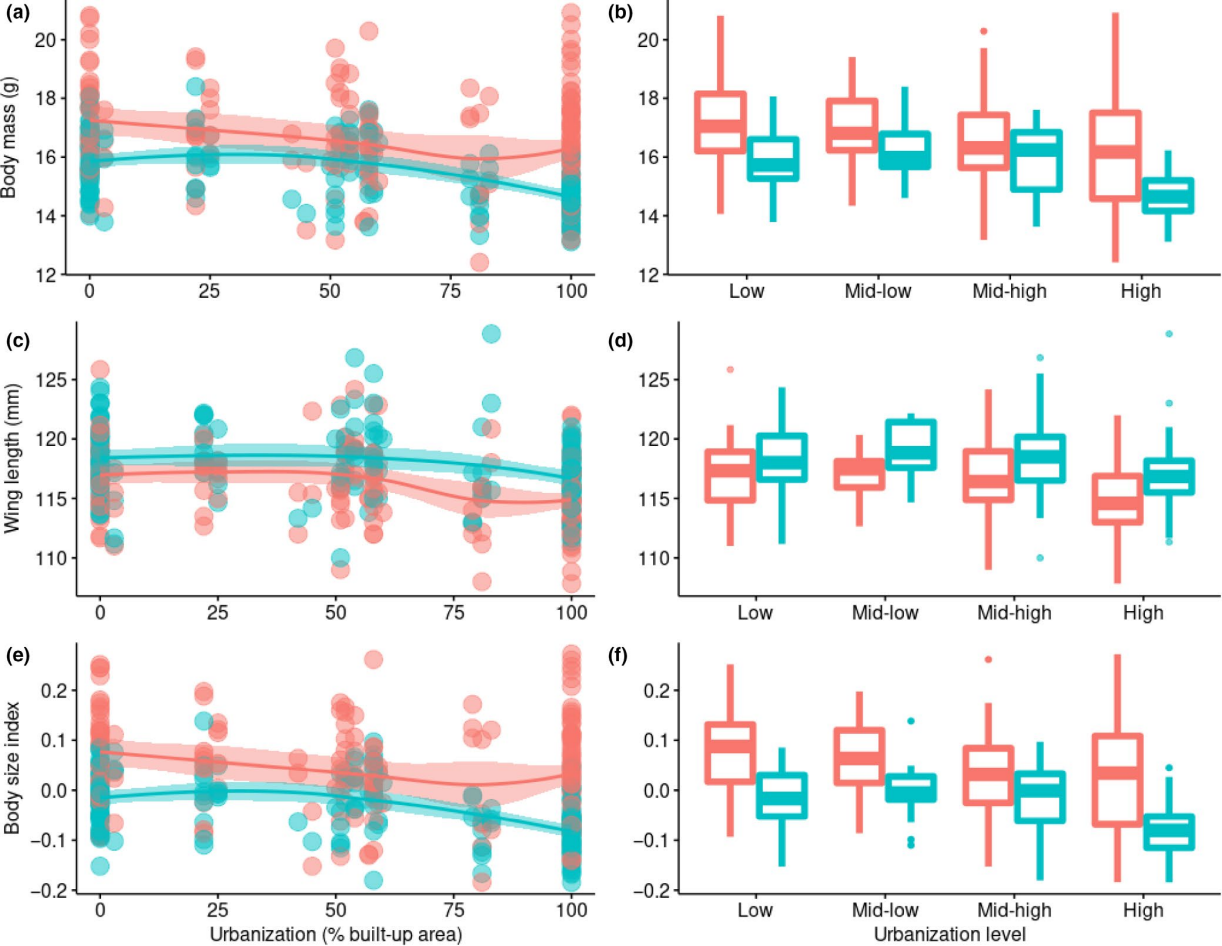
Differences in the body mass (a, b), wing length (c, d), and body size index (residuals from the regression between body mass and wing length) (e, f) of male (red color) and female (blue color) Barn Swallows *H. r. gutturalis* quantifying urbanization either as a continuous variable or categorical factor. In the scatter plots quantifying urbanization as a continuous variable (a, c, e), a local polynomial regression method (loess) was followed and 95% confidence intervals are represented as shaded areas. In the box plots quantifying urbanization as a categorical factor (Low, 0%–20%, 43 sites, *N* = 94; Mid‐low, 21%–40%, 17 sites, *N* = 29; Mid‐high, 41%–60%, 35 sites, *N* = 79; High, 61%–100%, 33 sites, *N* = 157) (b, d, f), central horizontal lines represent the median, thin horizontal lines represent the upper and lower quartiles, and vertical lines represent the maximum and minimum values. Outliers are shown in filled circles

The full model including both sexes showed no significant effect of urbanization on body mass, coded either as a continuous variable or as a categorical factor and regardless of whether we included wing length in the models or not (Table 1, Table A3). Body mass decreased significantly toward the south and the east, yet the relationship between body mass and longitude became nonsignificant in the models including wing length (Table A3). Females were on average significantly heavier than males (over 7%; Females, Mean ± *SD* = 16.58 ± 1.76 g; Males, Mean ± *SD* = 15.39 ± 1.10 g) (Figure 2a,b).

**Table 1:** Results of two linear mixed‐effect models fit by restricted maximum likelihood using log‐transformed body mass as a response variable, urbanization level, latitude, longitude, sampling date (*N* days from April 1st), and sex (female and male) as explanatory variables, and city and year as random factors

	Estimate	*SE*	*t*	*p*
Intercept	2.80	0.02	135.18	**<.001**
Urbanization	−0.004	0.008	−0.49	.63
Latitude	0.05	0.02	2.25	**.02**
Longitude	−0.04	0.02	−2.21	**.03**
Sampling date	−0.02	0.02	−1.19	.23
Sex (female vs. male)	−0.08	0.008	−9.98	**<.001**
Intercept	2.81	0.02	120.39	**<.001**
Low vs. Mid‐low	−0.005	0.03	−0.18	.85
Low vs. Mid‐high	<0.001	0.02	0.03	.98
Low vs. High	−0.01	0.02	−0.50	.62
Mid‐low vs. Mid‐high	0.005	0.02	0.23	.82
Mid‐low vs. High	−0.005	0.02	−0.23	.82
Mid‐high vs. High	−0.01	0.02	−0.58	.56
Latitude	0.05	0.02	2.28	**.02**
Longitude	−0.04	0.02	−2.17	**.03**
Sampling date	−0.03	0.02	−1.30	.19
Sex (female vs. male)	−0.08	0.01	−9.93	**<.001**

Note

We characterized urbanization as a continuous variable (up) and a categorical variable (Low, Mid‐low, Mid‐high, High) (down), respectively.

Significant effects are marked with bold.

In the model only including males, urbanization coded as a continuous variable correlated negatively with body mass (yet marginally significant, *p* = .05) (Table 2, Figure 2a). This relationship became significant in the model including wing length (*p* = .03) (Table A3). When urbanization was coded as a categorical factor, males in highly urbanized sites showed the lowest body mass. More specifically, males in highly urbanized sites showed lower body mass than individuals in low and mid‐high urbanized sites, and a nonsignificant tendency to have lower body mass than individuals in mid‐low urbanized sites (Figure 2b). The difference between mid‐low and highly urbanized sites became significant in the model including wing length (Table A4). As in the full model, males showed decreasing body mass toward the south and the east and, according to *t*‐values, these patterns were of similar magnitude to those related to urbanization. The models for females showed, conversely, no significant effects on body mass in any model (Table A5, Figure 2a,b). We only recorded a nonsignificant tendency of body mass to decrease with latitude in all models. Wing length and body mass correlated positively in all the previous models, while sampling date showed no significant effects in any model.

**Table 2:** Results of two linear mixed‐effect models fit by restricted maximum likelihood using log‐transformed male body mass as a response variable, urbanization level, latitude, longitude, sampling date (*N* days from April 1st), and sex (female and male) as explanatory variables, and city and year as random factors

	Estimate	*SE*	*t*	*p*
Intercept	2.73	0.02	176.73	**<.001**
Urbanization	−0.01	0.007	−1.96	.05
Latitude	0.04	0.01	2.51	**.01**
Longitude	−0.04	0.01	−3.61	**<.001**
Sampling date	−0.01	0.01	−0.73	.47
Intercept	2.74	0.01	203.58	**<.001**
Low vs. Mid‐low	−0.005	0.02	−0.22	.82
Low vs. Mid‐high	0.007	0.01	0.47	.64
Low vs. High	−0.04	0.02	−2.49	**.01**
Mid‐low vs. Mid‐high	0.01	0.02	0.57	.57
Mid‐low vs. High	−0.04	0.02	−1.77	.08
Mid‐high vs. High	−0.05	0.02	−3.04	**.002**
Latitude	0.04	0.01	2.86	**.004**
Longitude	−0.04	0.01	−3.40	**<.001**
Sampling date	−0.02	0.01	−1.45	.15

Note

We characterized urbanization as a continuous variable (up) and a categorical variable (Low, Mid‐low, Mid‐high, High) (down), respectively.

Significant effects are marked with bold.

### 3.2 Wing length

The full model including both sexes showed no significant effect of urbanization (either coded as continuous variable or categorical factor) on wing length. As with body mass, wing length decreased significantly toward south and east, yet in this case males had slightly longer wings on average than females (over 1.5%; Females, Mean ± *SD* = 115.96 ± 3.14 mm; Males, Mean ± *SD* = 117.79 ± 2.99 mm) (Table 3, Figure 2c,d).

**Table 3:** Results of two linear mixed‐effect models fit by restricted maximum likelihood using log‐transformed wing length as a response variable, urbanization level, latitude, longitude, sampling date (*N* days from April 1st), and sex (female and male) as explanatory variables, and city and year as random factors

	Estimate	*SE*	*t*	*p*
Intercept	4.75	0.002	2,130.71	**<.001**
Urbanization	−0.001	0.002	−0.76	.45
Latitude	0.01	0.004	2.93	**.003**
Longitude	−0.02	0.003	−6.59	**<.001**
Sampling date	−0.002	0.003	−0.78	.43
Sex (female vs. male)	0.01	0.002	5.92	**<.001**
Intercept	4.76	0.004	1,247.21	**<.001**
Low vs. Mid‐low	−0.01	0.006	−1.63	.10
Low vs. Mid‐high	−0.002	0.004	−0.56	.58
Low vs. High	−0.006	0.005	−1.26	.20
Mid‐low vs. Mid‐high	0.008	0.006	1.31	.19
Mid‐low vs. High	0.004	0.006	0.73	.46
Mid‐high vs. High	−0.003	0.004	−0.80	.43
Latitude	0.01	0.004	3.03	**.002**
Longitude	−0.02	0.003	−6.18	**<.001**
Sampling date	−0.003	0.004	−0.88	.38
Sex (female vs. male)	0.01	0.002	5.91	**<.001**

Note

We characterized urbanization as a continuous variable (up) and a categorical variable (Low, Mid‐low, Mid‐high, High) (down), respectively.

Significant effects are marked with bold.

The model only including males showed the same patterns than the full model (Table A6). Females had shorter wings in highly urbanized than low urbanized sites, yet this effect was not apparent when coding urbanization as a continuous variable (Table 4, Figure 2c,d). Regarding latitude and longitude, females showed the same patterns as for males and the full model. Sampling date showed no significant effect in any model.

**Table 4:** Results of two linear mixed‐effect models fit by restricted maximum likelihood using log‐transformed female wing length as a response variable, urbanization level, latitude, longitude, sampling date (*N* days from April 1st), and sex (female and male) as explanatory variables, and city and year as random factors

	Estimate	*SE*	*t*	*p*
Intercept	4.75	0.003	1,365.96	**<.001**
Urbanization	−0.004	0.003	−1.55	.12
Latitude	0.009	0.005	1.95	**.005**
Longitude	−0.02	0.003	−4.67	**<.001**
Sampling date	<0.001	0.005	0.07	.95
Intercept	4.76	0.005	962.85	**<.001**
Low vs. Mid‐low	−0.01	0.009	−1.40	.16
Low vs. Mid‐high	−0.004	0.006	−0.67	.51
Low vs. High	−0.01	0.006	−2.02	**.04**
Mid‐low vs. Mid‐high	0.008	0.008	0.98	.33
Mid‐low vs. High	<0.001	0.009	−0.06	.96
Mid‐high vs. High	−0.009	0.006	−1.53	.13
Latitude	0.01	0.005	2.35	**.02**
Longitude	−0.02	0.004	−4.59	**<.001**
Sampling date	−0.002	0.005	−0.35	.73

Note

We characterized urbanization as a continuous variable (up) and a categorical variable (Low, Mid‐low, Mid‐high, High) (down), respectively.

Significant effects are marked with bold.

### 3.3 Body size index

The full model including both sexes showed no effects of urbanization on the body size index, coded either as continuous variable or as categorical factor (Table A7). This proxy of body size decreased toward the south, yet showed no relationship with longitude, and was higher for females than for males.

The model including only males showed a similar pattern to that using body mass as dependent variable. Urbanization and the body size index correlated negatively (Table 5, Figure 2e). Furthermore, the body size index had the lowest scores in highly urbanized sites and this difference was significant with the rest of urbanization levels (Figure 2f). Males’ body size index decreased significantly toward the south and the east. Conversely, the model for females showed no significant effects (Table A8). Sampling date showed no significant effect in any model.

**Table 5:** Results of two linear mixed‐effect models fit by restricted maximum likelihood using body size index (body mass/wing length residual) of males as a response variable, urbanization level, latitude, longitude, sampling date (*N* days from April 1st), and sex (female and male) as explanatory variables, and city and year as random factors

	Estimate	*SE*	*t*	*p*
Intercept	−0.05	0.01	−3.18	**.001**
Urbanization	−0.02	0.007	−2.42	**.02**
Latitude	0.03	0.01	2.12	**.03**
Longitude	−0.03	0.01	−2.61	**.01**
Sampling date	−0.006	0.01	0.44	.66
Intercept	−0.03	0.01	−2.07	**.04**
Low vs. Mid‐low	0.001	0.02	0.05	.96
Low vs. Mid‐high	0.007	0.01	0.49	.62
Low vs. High	−0.05	0.02	−2.88	**.004**
Mid‐low vs. Mid‐high	0.006	0.02	0.29	.77
Mid‐low vs. High	−0.05	0.02	−2.29	**.02**
Mid‐high vs. High	−0.05	0.02	−3.44	**<.001**
Latitude	0.03	0.01	2.44	**.01**
Longitude	−0.02	0.01	−2.23	**.03**
Sampling date	−0.01	0.01	−1.19	.24

Note

We characterized urbanization as a continuous variable (up) and a categorical variable (Low, Mid‐low, Mid‐high, High) (down), respectively.

Significant effects are marked with bold.

## 4 DISCUSSION

In this study, after controlling for the effect of latitude and longitude across a very large geographical range (27.48° in longitude and 28.36° in latitude), we found that urbanization exerted a negative impact on body size of Barn Swallows. Nevertheless, this effect was only apparent when considering the sexes separately and affected different traits in males and females—body mass and wing length, respectively. This means that morphological divergence associated with urbanization acted in the same direction yet on different morphological traits in males and females (Caizergues et al., 2018; see also Przybylo et al., 2000, Millet et al., 2015). Urbanization drives considerable changes in many biotic and abiotic factors, which can affect both adults and their offspring (Heiss et al., 2009; Herrera‐Dueñas et al., 2017; Jiménez‐Peñuela et al., 2019; Liker et al., 2008; Ruiz et al., 2002), even at the embryonic stage (Bailly et al., 2016). In birds, these negative effects have been related to the heat‐island effect, habitat fragmentation and transformation, interspecific competition, and to the lack and low quality of food resources within urban areas (Heiss et al., 2009; Liker et al., 2008; Ruiz et al., 2002; Seress et al., 2020). However, these negative effects are not widespread among bird species (Bókony et al., 2012; Chamberlain et al., 2009; Giraudeau et al., 2014; Salmón et al., 2018) and, for instance, previous research has suggested that species with high dispersal capacity can evade the negative effects of urbanization (Merckx et al., 2018; Møller, 2009; Santini et al., 2019). Our results illustrated, therefore, several new facets of this issue. First, we found that urbanization seemed to exacerbate a natural geographical pattern of reduction in body size toward southern latitudes. Second, we found that these negative effects could also be apparent in species with very high mobility, such as Barn Swallows. Finally, we found that urbanization and geographical variation could have sex‐dependent effects, since in our sample different traits were affected to a different extent by urbanization, latitude, and longitude in each sex.

We also recorded a strong impact of geography on body size variation, with similar patterns, yet also sex‐dependent, for body mass and wing length. Western and Northern males were heavier than Eastern and Southern ones, either considering body mass or the body size index, which seems consistent across life stages (Pagani‐Núñez et al., 2016). The same pattern was apparent for both sexes regarding wing length. Latitudinal variation in body size is often interpreted as a manifestation of Bergmann's rule, which is a classic and popular theory that explains spatial variation in body size across species and populations (Ashton, 2002). Animals in cold areas at high latitudes or altitudes are usually larger than individuals of the same species in warmer areas (Meiri & Dayan, 2003). Although this effect could have been ameliorated in Barn Swallows, which are a migratory species and thus may not necessarily experience strong climatic constraints to body size development (Olson et al., 2009), we recorded a clear pattern of decreasing body size toward warmer geographical areas. In China, there is significant climatic variation across geographical regions, with increasing temperature and humidity from West to East and North to South (Domrös & Peng, 2012). The combined effect of climatic variation and high urbanization levels in East and South China, which has likely fostered the urban heat‐island effect in those areas, contributed to a similar extent that urbanization to shape body size variation of male Barn Swallows across this broad country.

Interestingly, females showed a slightly different pattern than males. Females were heavier and had shorter wings than males. Moreover, female wing length rather than body mass responded to urbanization. A combination of factors may be required to explain this sexual difference. On the one hand, males usually have to spend more energy on sexual displays and nest defense, so particularly the smallest individuals from southern populations could be more sensitive to the typically adverse environmental conditions associated with urbanization (Møller & Szép, 2002; Saino et al., 2003). On the other hand, females in southern populations could display shorter migrations as, for example, in Common Chiffchaffs *Phylloscopus collybita* (Catry et al., 2005), particularly in urban areas, which could influence wing morphology. This link between migratory behavior and wing morphology is usually referred to as Seebohm's rule. This rule has been broadly discussed using Blackbirds *Turdus merula* as model, with such research obtaining conflictive results (see, e.g., Evans et al., 2009; Saccavino et al., 2018). Constraints to development associated with an urban lifestyle, or any potential advantages of having a reduced body size (Caizergues et al., 2018), acted here on different traits. To what extent body size variation can be associated to either negative effects of urbanization, or potential selective advantages, remains to be explored.

To conclude, body size is an important trait in birds, being directly related to survival and fitness (Liu et al., 2018; Møller & Szép, 2002; Moreno‐Rueda, 2011; Price & Liou, 1989; Saether, 1989). As the result of the trade‐off between predation and starvation risk (Lima, 1986), body size can be affected by various biotic and abiotic factors. Here we found that Barn Swallow in urban areas of East and South China showed the lowest body size across a vast geographical area, suggesting that urbanization may make these populations the most vulnerable in face of current landscape and climate change.

## CONFLICT OF INTEREST

There is no conflict of interest to declare.

## AUTHOR CONTRIBUTION

**Yanyan Zhao:** Conceptualization (supporting); Data curation (equal); Formal analysis (lead); Investigation (lead); Writing‐original draft (equal); Writing‐review & editing (equal). **Yu Liu:** Conceptualization (supporting); Data curation (equal); Formal analysis (supporting); Investigation (lead); Methodology (equal); Writing‐original draft (equal); Writing‐review & editing (equal). **Elizabeth S. C. Scordato:** Investigation (equal); Methodology (equal); Writing‐original draft (equal); Writing‐review & editing (equal). **Myung‐Bok Lee:** Formal analysis (lead); Methodology (supporting); Writing‐original draft (supporting); Writing‐review & editing (supporting). **Xiaoying Xing:** Conceptualization (supporting); Funding acquisition (equal); Writing‐original draft (supporting); Writing‐review & editing (supporting). **Xinyuan Pan:** Funding acquisition (supporting); Investigation (supporting); Writing‐original draft (supporting); Writing‐review & editing (supporting). **Yang Liu:** Conceptualization (supporting); Formal analysis (supporting); Funding acquisition (supporting); Writing‐original draft (supporting); Writing‐review & editing (supporting). **Rebecca J. Safran:** Conceptualization (supporting); Formal analysis (supporting); Funding acquisition (equal); Methodology (equal); Writing‐original draft (supporting); Writing‐review & editing (equal). **Emilio Pagani‐Núñez:** Conceptualization (lead); Data curation (supporting); Formal analysis (equal); Funding acquisition (equal); Investigation (supporting); Writing‐original draft (equal); Writing‐review & editing (equal).

## Data Availability

The dataset used in this article is archived at Dryad (https://doi.org/10.5061/dryad.h70rxwdh1).

## 1

**Table A1:** Number of sampling sites and individuals across the different urbanization levels

Urbanization level	Sampling sites	Average built‐up proportion	Females	Males
Low	43	0.35	39	55
Mid‐low	17	23.06	14	15
Mid‐high	35	54.63	42	37
High	33	94.12	80	77

Note

Numbers for males and females separately is also provided.

**Table A2:** Pearson correlation of geographic and climatic variables

	Latitude	Longitude	Annual temperature	Annual precipitation
Latitude	1			
Longitude	−0.865	1		
Annual temperature	0.715	−0.362	1	
Annual precipitation	0.845	−0.919	0.302	1

**Table A3:** Results of two linear mixed‐effect models fit by restricted maximum likelihood using log‐transformed body mass as a response variable, urbanization level, log‐transformed wing length, latitude, longitude, sampling date (*N* days from April 1st), and sex (female and male) as explanatory variables, and city and year as random factors

	Estimate	*SE*	*t*	*p*
Intercept	0.16	0.86	0.18	.86
Urbanization	−0.004	0.008	−0.58	.56
Wing length	1.28	0.42	3.06	**002**
Latitude	0.04	0.02	2.17	**.03**
Longitude	−0.03	0.02	−1.76	.08
Sampling date	−0.02	0.02	−1.20	.23
Sex (female vs. male)	−0.09	0.008	−10.51	**<.001**
Intercept	0.18	0.87	0.21	.83
Low vs. Mid‐low	−0.001	0.02	−0.03	.98
Low vs. Mid‐high	−0.001	0.02	−0.05	.96
Low vs. High	−0.009	0.02	−0.48	.63
Mid‐low vs. Mid‐high	<0.001	0.02	−0.002	.99
Mid‐low vs. High	−0.008	0.02	−0.38	.70
Mid‐high vs. High	−0.008	0.02	−0.48	.63
Wing length	1.27	0.42	3.02	**.003**
Latitude	0.04	0.02	2.15	**.03**
Longitude	−0.03	0.02	−1.69	.09
Sampling date	−0.02	0.02	−1.28	.20
Sex (female vs. male)	−0.09	0.008	−10.46	**<.001**

Note

The two models used urbanization level as a continuous variable and a categorical variable (Low, Mid‐low, Mid‐high, High), respectively.

Significant effects are marked with bold.

**Table A4:** Results of two linear mixed‐effect models fit by restricted maximum likelihood using log‐transformed male body mass as a response variable, urbanization level, log‐transformed wing length, latitude, longitude, sampling date (*N* days from April 1st), and sex (female and male) as explanatory variables, and city and year as random factors

	Estimate	*SE*	*t*	*p*
Intercept	0.58	0.91	0.64	.52
Urbanization	−0.02	0.007	−2.22	**.03**
Wing length	1.04	0.44	2.36	**.02**
Latitude	0.03	0.01	2.29	**.02**
Longitude	−0.03	0.01	−2.95	**.003**
Sampling date	−0.008	0.01	−0.58	.56
Intercept	0.48	0.90	0.53	.60
Low vs. Mid‐low	−0.001	0.02	−0.05	.96
Low vs. Mid‐high	0.007	0.01	0.49	.62
Low vs. High	−0.04	0.02	−2.72	**.007**
Mid‐low vs. Mid‐high	0.008	0.02	0.4	.69
Mid‐low vs. High	−0.04	0.02	−2.08	**.04**
Mid‐high vs. High	−0.05	0.02	−3.28	**.001**
Wing length	1.10	0.44	2.51	**.01**
Latitude	0.04	0.01	2.59	**.009**
Longitude	−0.03	0.01	−2.60	**.009**
Sampling date	−0.02	0.01	−1.30	.19

Note

The two models used urbanization level as a continuous variable and a categorical variable (Low, Mid‐low, Mid‐high, High), respectively.

Significant effects are marked with bold.

**Table A5:** Results of four linear mixed‐effect models fit by restricted maximum likelihood using log‐transformed female body mass as a response variable, urbanization level, latitude, longitude, sampling date (*N* days from April 1st), and sex (female and male) as explanatory variables, and city and year as random factors

	Estimate	*SE*	*t*	*p*
Intercept	2.80	0.02	121.25	**<.001**
Urbanization	0.001	0.01	0.06	.95
Latitude	0.06	0.03	1.82	.07
Longitude	−0.03	0.02	−1.24	.21
Sampling date	−0.04	0.03	−1.29	.20
Intercept	2.80	0.03	84.02	**<.001**
Low vs. Mid‐low	−0.01	0.04	−0.35	.72
Low vs. Mid‐high	−0.01	0.03	−0.38	.72
Low vs. High	−0.003	0.03	0.11	.91
Mid‐low vs. Mid‐high	0.004	0.04	0.10	.92
Mid‐low vs. High	0.02	0.04	0.50	.62
Mid‐high vs. High	0.01	0.03	0.54	.59
Latitude	0.06	0.03	1.76	.08
Longitude	−0.03	0.02	−1.30	.19
Sampling date	−0.03	0.03	−1.15	.25
Intercept	−0.48	1.40	−0.34	.73
Urbanization	0.002	0.01	0.19	.85
Wing length	1.59	0.68	2.34	**.02**
Latitude	0.05	0.03	1.78	.07
Longitude	−0.02	0.02	−0.83	.41
Sampling date	−0.04	0.03	−1.46	.14
Intercept	−0.58	1.42	−0.41	.68
Low vs. Mid‐low	−0.01	0.04	−0.24	.81
Low vs. Mid‐high	−0.01	0.03	−0.44	.66
Low vs. High	0.01	0.03	0.32	.75
Mid‐low vs. Mid‐high	−0.003	0.04	−0.08	.94
Mid‐low vs. High	0.02	0.04	0.55	.58
Mid‐high vs. High	0.02	0.03	0.85	.40
Wing length	1.64	0.69	2.39	**.02**
Latitude	0.05	0.03	1.65	.10
Longitude	−0.02	0.02	−0.89	.37
Sampling date	−0.03	0.03	−1.22	.22

Note

The two models including or not including log‐transformed wing length as another explanatory variable both included urbanization level as a continuous variable and a categorical variable (Low, Mid‐low, Mid‐high, High), respectively.

Significant effects are marked with bold.

**Table A6:** Results of two linear mixed‐effect models fit by restricted maximum likelihood using log‐transformed male wing length as a response variable, urbanization level, latitude, longitude, sampling date (*N* days from April 1st), and sex (female and male) as explanatory variables, and city and year as random factors

	Estimate	*SE*	*t*	*p*
Intercept	4.77	0.002	2,823.23	**<.001**
Urbanization	<0.001	0.002	0.05	.96
Latitude	0.01	0.003	3.44	**.001**
Longitude	−0.02	0.002	−7.75	**<.001**
Sampling date	−0.004	0.003	−1.30	.20
Intercept	4.77	0.004	1,316.87	**<.001**
Low vs. Mid‐low	−0.007	0.007	−0.96	.34
Low vs. Mid‐high	−0.004	0.005	−0.79	.43
Low vs. High	<0.001	0.005	−0.04	.97
Mid‐low vs. Mid‐high	0.003	0.007	0.37	.71
Mid‐low vs. High	0.007	0.007	0.90	.37
Mid‐high vs. High	0.004	0.005	0.75	.45
Latitude	0.01	0.004	3.20	**.001**
Longitude	−0.02	0.003	−6.91	**<.001**
Sampling date	−0.004	0.004	−1.17	.24

Note

The two models used urbanization level as a continuous variable and a categorical variable (Low, Mid‐low, Mid‐high, High), respectively.

Significant effects are marked with bold.

**Table A7:** Results of two linear mixed‐effect models fit by restricted maximum likelihood using body size index (body mass/wing length residual) as a response variable, urbanization level, latitude, longitude, sampling date (*N* days from April 1st), and sex (female and male) as explanatory variables, and city and year as random factors

	Estimate	*SE*	*t*	*p*
Intercept	0.24	0.17	1.38	.17
Urbanization	<0.001	<0.001	−0.64	.52
Latitude	0.005	0.002	2.14	**.03**
Longitude	−0.003	0.002	−1.57	.12
Sampling date	−0.001	0.001	−1.19	.23
Sex (female vs. male)	−0.09	0.008	−11.41	**<.001**
Intercept	0.046	0.02	2.10	**.04**
Low vs. Mid‐low	0.001	0.02	0.02	.98
Low vs. Mid‐high	−0.002	0.02	−0.10	.92
Low vs. High	−0.009	0.02	−0.49	.63
Mid‐low vs. Mid‐high	−0.002	0.02	−0.10	.92
Mid‐low vs. High	−0.01	0.02	−0.45	.65
Mid‐high vs. High	−0.008	0.02	−0.45	.65
Latitude	0.04	0.02	2.09	**.04**
Longitude	−0.02	0.02	−1.49	.14
Sampling date	−0.02	0.02	−1.25	.21
Sex (female vs. male)	−0.09	0.008	−11.37	**<.001**

Note

The two models used urbanization level as a continuous variable and a categorical variable (Low, Mid‐low, Mid‐high, High), respectively. Significant effects are marked with bold.

**Table A8:** Results of two linear mixed‐effect models fit by restricted maximum likelihood using body size index (body mass/wing length residual) of females as a response variable, urbanization level, latitude, longitude, sampling date (*N* days from April 1st), and sex (female and male) as explanatory variables, and city and year as random factors

	Estimate	*SE*	*t*	*p*
Intercept	0.04	0.02	2.30	**.02**
Urbanization	0.002	0.01	0.21	.84
Latitude	0.05	0.03	1.79	.07
Longitude	−0.02	0.02	−0.79	.43
Sampling date	−0.04	0.03	−1.49	.14
Intercept	0.04	0.03	1.32	.19
Low vs. Mid‐low	−0.009	0.04	−0.22	.83
Low vs. Mid‐high	−0.01	0.03	−0.45	.65
Low vs. High	0.01	0.03	0.36	.72
Mid‐low vs. Mid‐high	−0.004	0.04	−0.10	.92
Mid‐low vs. High	0.02	0.04	0.55	.58
Mid‐high vs. High	0.02	0.03	0.90	.37
Latitude	0.05	0.03	1.64	.10
Longitude	−0.02	0.02	−0.86	.39
Sampling date	−0.03	0.03	−1.23	.22

Note

The two models used urbanization level as a continuous variable and a categorical variable (Low, Mid‐low, Mid‐high, High), respectively.

Significant effects are marked with bold.

## References

[ece37088-bib-0001] Alberti, M. (2015). Eco‐evolutionary dynamics in an urbanizing planet. Trends in Ecology and Evolution, 30, 114–126. 10.1016/j.tree.2014.11.007 25498964

[ece37088-bib-0002] Allen, C. R. , Garmestani, A. S. , Havlicek, T. D. , Marquet, P. A. , Peterson, G. D. , Restrepo, C. , Stow, C. A. , & Weeks, B. E. (2006). Patterns in body mass distributions: Sifting among alternative hypotheses. Ecology Letters, 9, 630–643. 10.1111/j.1461-0248.2006.00902.x 16643307

[ece37088-bib-0003] Andrew, S. C. , Awasthy, M. , Griffith, A. D. , Nakagawa, S. , & Griffith, S. C. (2018). Clinal variation in avian body size is better explained by summer maximum temperatures during development than by cold winter temperatures. The Auk, 135, 206–217. 10.1642/AUK-17-129.1

[ece37088-bib-0004] Ashton, K. G. (2002). Patterns of within‐species body size variation of birds: Strong evidence for Bergmann’s rule. Global Ecology and Biogeography, 11, 505–523. 10.1046/j.1466-822X.2002.00313.x

[ece37088-bib-0005] Atkinson, D. (1994). Temperature and organism size: A biological law for ectotherms? Advances in Ecological Research, 25, 1–58.

[ece37088-bib-0006] Bailly, J. , Scheifler, R. , Berthe, S. , Clément‐Demange, V.‐A. , Leblond, M. , Pasteur, B. , & Faivre, B. (2016). From eggs to fledging: Negative impact of urban habitat on reproduction in two tit species. Journal of Ornithology, 157, 377–392. 10.1007/s10336-015-1293-3

[ece37088-bib-0007] Barr, D. J. , Levy, R. , Scheepers, C. , & Tily, H. J. (2013). Random effects structure for confirmatory hypothesis testing: Keep it maximal. Journal of Memory and Language, 68, 255–278. 10.1016/j.jml.2012.11.001 PMC3881361 24403724

[ece37088-bib-0008] Bates, D. , Mächler, M. , Bolker, B. , & Walker, S. (2015). Fitting linear mixed‐effects models using lme4. Journal of Statistical Software, 67, 1‐48. 10.18637/jss.v067.i01

[ece37088-bib-0009] Bivand, R. S. , & Wong, D. W. S. (2018). Comparing implementations of global and local indicators of spatial association. TEST, 27, 716–748. 10.1007/s11749-018-0599-x

[ece37088-bib-0010] Bókony, V. , Seress, G. , Nagy, S. , Lendvai, Á. Z. , & Liker, A. (2012). Multiple indices of body condition reveal no negative effect of urbanization in adult house sparrows. Landscape and Urban Planning, 104, 75–84. 10.1016/j.landurbplan.2011.10.006

[ece37088-bib-0011] Caizergues, A. E. , Grégoire, A. , & Charmantier, A. (2018). Urban versus forest ecotypes are not explained by divergent reproductive selection. Proceedings of the Royal Society B: Biological Sciences, 285, 20180261 10.1098/rspb.2018.0261 PMC6053928 30051819

[ece37088-bib-0012] Catry, P. , Lecoq, M. , Araújo, A. , Conway, G. , Felgueiras, M. , King, J. M. B. , Rumsey, S. , Salima, H. , & Tenreiro, P. (2005). Differential migration of chiffchaffs *Phylloscopus collybita* and *P. ibericus* in Europe and Africa. Journal of Avian Biology, 36, 184–190. 10.1111/j.0908-8857.2005.03445.x

[ece37088-bib-0013] Chamberlain, D. E. , Cannon, A. R. , Toms, M. P. , Leech, D. I. , Hatchwell, B. J. , & Gaston, K. J. (2009). Avian productivity in urban landscapes: A review and meta‐analysis. Ibis, 151, 1–18. 10.1111/j.1474-919X.2008.00899.x

[ece37088-bib-0014] Domrös, M. , & Peng, G. (2012). The climate of China. Springer Science & Business Media.

[ece37088-bib-0015] Dor, R. , Safran, R. J. , Sheldon, F. H. , Winkler, D. W. , & Lovette, I. J. (2010). Phylogeny of the genus Hirundo and the Barn Swallow subspecies complex. Molecular Phylogenetics and Evolution, 56, 409–418. 10.1016/j.ympev.2010.02.008 20152914

[ece37088-bib-0016] Evans, K. L. , Chamberlain, D. E. , Hatchwell, B. J. , Gregory, R. D. , & Gaston, K. J. (2011). What makes an urban bird? Global Change Biology, 17, 32–44. 10.1111/j.1365-2486.2010.02247.x

[ece37088-bib-0017] Evans, K. L. , Gaston, K. J. , Sharp, S. P. , McGowan, A. , & Hatchwell, B. J. (2009). The effect of urbanisation on avian morphology and latitudinal gradients in body size. Oikos, 118, 251–259. 10.1111/j.1600-0706.2008.17092.x

[ece37088-bib-0018] Fox, J. , & Weisberg, S. (2018). An R companion to applied regression. Sage Publications.

[ece37088-bib-0019] Giraudeau, M. , Mousel, M. , Earl, S. , & McGraw, K. (2014). Parasites in the city: Degree of urbanization predicts poxvirus and coccidian infections in house finches (*Haemorhous mexicanus*). PLoS One, 9, e86747 10.1371/journal.pone.0086747 24503816 PMC3913573

[ece37088-bib-0020] Heiss, R. S. , Clark, A. B. , & McGowan, K. J. (2009). Growth and nutritional state of American Crow nestlings vary between urban and rural habitats. Ecological Applications, 19, 829–839. 10.1890/08-0140.1 19544727

[ece37088-bib-0021] Hendry, A. P. , Farrugia, T. J. , & Kinnison, M. T. (2008). Human influences on rates of phenotypic change in wild animal populations. Molecular Ecology, 17, 20–29. 10.1111/j.1365-294X.2007.03428.x 18173498

[ece37088-bib-0022] Herrera‐Dueñas, A. , Pineda‐Pampliega, J. , Antonio‐García, M. T. , & Aguirre, J. I. (2017). The influence of urban environments on oxidative stress balance: A case study on the house sparrow in the Iberian Peninsula. Frontiers in Ecology and Evolution, 5, 106 10.3389/fevo.2017.00106

[ece37088-bib-0023] Jiménez‐Peñuela, J. , Ferraguti, M. , Martínez‐de la Puente, J. , Soriguer, R. , & Figuerola, J. (2019). Urbanization and blood parasite infections affect the body condition of wild birds. Science of the Total Environment, 651, 3015–3022. 10.1016/j.scitotenv.2018.10.203 30463151

[ece37088-bib-0024] Johnson, M. T. J. , & Munshi‐South, J. (2017). Evolution of life in urban environments. Science, 358, eaam8327 10.1126/science.aam8327 29097520

[ece37088-bib-0025] Liker, A. , Papp, Z. , Bókony, V. , & Lendvai, Á. Z. (2008). Lean birds in the city: Body size and condition of house sparrows along the urbanization gradient. Journal of Animal Ecology, 77, 789–795. 10.1111/j.1365-2656.2008.01402.x 18479344

[ece37088-bib-0026] Lima, S. L. (1986). Predation risk and unpredictable feeding conditions: Determinants of body mass in birds. Ecology, 67, 377–385. 10.2307/1938580

[ece37088-bib-0027] Lin, C.‐H. , Hsu, C.‐Y. , & Lin, J.‐Y. (2015). Using daily light integral concept to construct the ecological plant design strategy of urban landscape. International Scholarly and Scientific Research and Innovation, 9, 891–897.

[ece37088-bib-0028] Liu, Y. , Scordato, E. S. C. , Safran, R. , & Evans, M. (2018). Ventral colour, not tail streamer length, is associated with seasonal reproductive performance in a Chinese population of Barn Swallows (Hirundo rustica gutturalis). Journal of Ornithology, 159, 675–685. 10.1007/s10336-018-1555-y

[ece37088-bib-0029] Liu, Y. , Scordato, E. S. , Zhang, Z. , Evans, M. , & Safran, R. J. (2020). Analysing phenotypic variation in barn swallows (*Hirundo rustica*) across China to assess subspecies status. Biological Journal of the Linnean Society, 131, 319–331. 10.1093/biolinnean/blaa112

[ece37088-bib-0030] McKinney, M. L. (2008). Effects of urbanization on species richness: A review of plants and animals. Urban Ecosystems, 11, 161–176. 10.1007/s11252-007-0045-4

[ece37088-bib-0031] Meiri, S. , & Dayan, T. (2003). On the validity of Bergmann’s rule. Journal of Biogeography, 30, 331–351. 10.1046/j.1365-2699.2003.00837.x

[ece37088-bib-0032] Merckx, T. , Souffreau, C. , Kaiser, A. , Baardsen, L. F. , Backeljau, T. , Bonte, D. , Brans, K. I. , Cours, M. , Dahirel, M. , Debortoli, N. , De Wolf, K. , Engelen, J. M. T. , Fontaneto, D. , Gianuca, A. T. , Govaert, L. , Hendrickx, F. , Higuti, J. , Lens, L. , Martens, K. , … Van Dyck, H. (2018). Body‐size shifts in aquatic and terrestrial urban communities. Nature, 558, 113–116. 10.1038/s41586-018-0140-0 29795350

[ece37088-bib-0033] Millet, A. , Pelletier, F. , Bélisle, M. , & Garant, D. (2015). Patterns of fluctuating selection on morphological and reproductive traits in female tree swallow (*Tachycineta bicolor*). Evolutionary Biology, 42, 349–358. 10.1007/s11692-015-9333-8

[ece37088-bib-0034] Møller, A. P. (2009). Successful city dwellers: A comparative study of the ecological characteristics of urban birds in the Western Palearctic. Oecologia, 159, 849–858. 10.1007/s00442-008-1259-8 19139922

[ece37088-bib-0035] Møller, A. P. , & Szép, T. (2002). Survival rate of adult Barn Swallow *Hirundo rustica* in relation to sexual selection and reproduction. Ecology, 83, 2220–2228.

[ece37088-bib-0036] Moreno‐Rueda, G. (2011). Trade‐off between immune response and body mass in wintering house sparrows (*Passer domesticus*). Ecological Research, 26, 943–947. 10.1007/s11284-011-0848-x

[ece37088-bib-0037] Newbold, T. , Hudson, L. N. , Hill, S. L. L. , Contu, S. , Lysenko, I. , Senior, R. A. , Börger, L. , Bennett, D. J. , Choimes, A. , Collen, B. , Day, J. , De Palma, A. , Díaz, S. , Echeverria‐Londoño, S. , Edgar, M. J. , Feldman, A. , Garon, M. , Harrison, M. L. K. , Alhusseini, T. , … Purvis, A. (2015). Global effects of land use on local terrestrial biodiversity. Nature, 520, 45–50. 10.1038/nature14324 25832402

[ece37088-bib-0038] Olson, V. A. , Davies, R. G. , Orme, C. D. L. , Thomas, G. H. , Meiri, S. , Blackburn, T. M. , Gaston, K. J. , Owens, I. P. F. , & Bennett, P. M. (2009). Global biogeography and ecology of body size in birds. Ecology Letters, 12, 249–259. 10.1111/j.1461-0248.2009.01281.x 19245587

[ece37088-bib-0039] Pagani‐Núñez, E. , He, C. , Li, B. , Li, M. , He, R. , Jiang, A. , & Goodale, E. (2016). The breeding ecology of the barn swallow *Hirundo rustica gutturalis* in South China. Journal of Tropical Ecology, 32, 260–263. 10.1017/S0266467416000201

[ece37088-bib-0040] Peng, S. , Ding, Y. , Liu, W. , & Li, Z. (2019). 1 km monthly temperature and precipitation dataset for China from 1901 to 2017. Earth System Science Data, 11, 1931–1946. 10.5194/essd-11-1931-2019

[ece37088-bib-0041] Pollock, C. J. , Capilla‐Lasheras, P. , McGill, R. A. , Helm, B. , & Dominoni, D. M. (2017). Integrated behavioural and stable isotope data reveal altered diet linked to low breeding success in urban‐dwelling blue tits (*Cyanistes caeruleus*). Scientific Reports, 7, 1–14. 10.1038/s41598-017-04575-y 28694437 PMC5503996

[ece37088-bib-0042] Price, T. , & Liou, L. (1989). Selection on clutch size in birds. The American Naturalist, 134, 950–959. 10.1086/285023

[ece37088-bib-0043] Przybylo, R. , Sheldon, B. C. , & Merilä, J. (2000). Patterns of natural selection on morphology of male and female collared flycatchers (*Ficedula albicollis*). Biological Journal of the Linnean Society, 69, 213–232. 10.1111/j.1095-8312.2000.tb01199.x

[ece37088-bib-0044] R Core Team (2020). R: A language and environment for statistical computing. R Foundation for Statistical Computing.

[ece37088-bib-0045] Riyahi, S. , Hammer, Ø. , Arbabi, T. , Sánchez, A. , Roselaar, C. S. , Aliabadian, M. , & Sætre, G. P. (2013). Beak and skull shapes of human commensal and non‐commensal house sparrows Passer domesticus. BMC Evolutionary Biology, 13, 200 10.1186/1471-2148-13-200 24044497 PMC3850535

[ece37088-bib-0046] Ruiz, G. , Rosenmann, M. , Novoa, F. F. , & Sabat, P. (2002). Hematological parameters and stress index in Rufous‐collared Sparrows dwelling in urban environments. The Condor, 104, 162.

[ece37088-bib-0047] Saccavino, E. , Krämer, J. , Klaus, S. , & Tietze, D. T. (2018). Does urbanization affect wing pointedness in the Blackbird *Turdus merula*? Journal of Ornithology, 159, 1043–1051. 10.1007/s10336-018-1575-7

[ece37088-bib-0048] Saether, B.‐E. (1989). Survival rates in relation to body weight in European birds. Ornis Scandinavica, 20, 13–21. 10.2307/3676702

[ece37088-bib-0049] Safran, R. J. , Vortman, Y. , Jenkins, B. R. , Hubbard, J. K. , Wilkins, M. R. , Bradley, R. J. , & Lotem, A. (2016). The maintenance of phenotypic divergence through sexual selection: An experimental study in barn swallows *Hirundo rustica* . Evolution, 70, 2074–2084. 10.1111/evo.13014 27436630

[ece37088-bib-0050] Saino, N. , Romano, M. , Sacchi, R. , Ninni, P. , Galeotti, P. , & Møller, A. P. (2003). Do male Barn Swallows (*Hirundo rustica*) experience a trade‐off between the expression of multiple sexual signals? Behavioral Ecology and Sociobiology, 54, 465–471. 10.1007/s00265-003-0642-z

[ece37088-bib-0051] Salmón, P. , Stroh, E. , Herrera‐Dueñas, A. , von Post, M. , & Isaksson, C. (2018). Oxidative stress in birds along a NOx and urbanisation gradient: An interspecific approach. Science of the Total Environment, 622, 635–643. 10.1016/j.scitotenv.2017.11.354 29223087

[ece37088-bib-0052] Santini, L. , González‐Suárez, M. , Russo, D. , Gonzalez‐Voyer, A. , von Hardenberg, A. , & Ancillotto, L. (2019). One strategy does not fit all: Determinants of urban adaptation in mammals. Ecology Letters, 22, 365–376. 10.1111/ele.13199 30575254 PMC7379640

[ece37088-bib-0053] Scheffers, B. R. , De Meester, L. , Bridge, T. C. L. , Hoffmann, A. A. , Pandolfi, J. M. , Corlett, R. T. , Butchart, S. H. M. , Pearce‐Kelly, P. , Kovacs, K. M. , Dudgeon, D. , Pacifici, M. , Rondinini, C. , Foden, W. B. , Martin, T. G. , Mora, C. , Bickford, D. , & Watson, J. E. M. (2016). The broad footprint of climate change from genes to biomes to people. Science, 354, aaf7671 10.1126/science.aaf7671 27846577

[ece37088-bib-0054] Scordato, E. S. , & Safran, R. J. (2014). Geographic variation in sexual selection and implications for speciation in the Barn Swallow. Avian Research, 5, 8 10.1186/s40657-014-0008-4

[ece37088-bib-0055] Seress, G. , Sándor, K. , Evans, K. L. , & Liker, A. (2020). Food availability limits avian reproduction in the city: An experimental study on great tits Parus major. Journal of Animal Ecology, 89, 1570–1580. 10.1111/1365-2656.13211 32419138

[ece37088-bib-0056] Sol, D. , González‐Lagos, C. , Moreira, D. , Maspons, J. , & Lapiedra, O. (2014). Urbanisation tolerance and the loss of avian diversity. Ecology Letters, 17, 942–950. 10.1111/ele.12297 24835452

[ece37088-bib-0057] Sol, D. , Trisos, C. , Múrria, C. , Jeliazkov, A. , González‐Lagos, C. , Pigot, A. L. , Ricotta, C. , Swan, C. M. , Tobias, J. A. , & Pavoine, S. (2020). The worldwide impact of urbanisation on avian functional diversity. Ecology Letters, 23, 962–972. 10.1111/ele.13495 32266768

[ece37088-bib-0058] Szulkin, M. , Garroway, C. J. , Corsini, M. , Kotarba, A. Z. , & Dominoni, D. (2020). How to quantify urbanization when testing for urban evolution? In Szulkin M., Munshi‐South J., & Charmantier A. (Eds.), Urban evolutionary biology (pp. 13–35). Oxford University Press.

[ece37088-bib-0059] Tuanmu, M.‐N. , & Jetz, W. (2014). A global 1‐km consensus land‐cover product for biodiversity and ecosystem modelling: Consensus land cover. Global Ecology and Biogeography, 23, 1031–1045. 10.1111/geb.12182

[ece37088-bib-0060] Zhang, S. , Lei, F. , Liu, S. , Li, D. , Chen, C. , & Wang, P. (2011). Variation in baseline corticosterone levels of Tree Sparrow (*Passer montanus*) populations along an urban gradient in Beijing, China. Journal of Ornithology, 152, 801–806. 10.1007/s10336-011-0663-8

[ece37088-bib-0061] Zhou, D. , Zhang, L. , Hao, L. , Sun, G. , Liu, Y. , & Zhu, C. (2016). Spatiotemporal trends of urban heat island effect along the urban development intensity gradient in China. Science of the Total Environment, 544, 617–626. 10.1016/j.scitotenv.2015.11.168 26674691

[ece37088-bib-0062] Zhou, L. , Dickinson, R. E. , Tian, Y. , Fang, J. , Li, Q. , Kaufmann, R. K. , Tucker, C. J. , & Myneni, R. B. (2004). Evidence for a significant urbanization effect on climate in China. Proceedings of the National Academy of Sciences of the United States of America, 101, 9540–9544. 10.1073/pnas.0400357101 15205480 PMC470710

