# CORRIGENDUM

**DOI:** 10.1002/ece3.7285

**Published:** 2021-04-02

In “The impact of urbanization on body size of Barn Swallows *Hirundo rustica gutturalis,*” which was published in volume 11 issue 1, January 2021, the colors mentioned for male and female in the legend of Figure 2 were wrong. The start of the legend in the published version reads:

Differences in the body mass (a, b), wing length (c, d), and body size index (residuals from the regression between body mass and wing length) (e, f) of male (red color) and female (blue color) Barn Swallows *H. r. gutturalis* quantifying urbanization either as a continuous variable or categorical factor.

The correct statement should be:

Differences in the body mass (a, b), wing length (c, d), and body size index (residuals from the regression between body mass and wing length) (e, f) of male (blue color) and female (red color) Barn Swallows *H. r. gutturalis* quantifying urbanization either as a continuous variable or categorical factor.

REFERENCE

Zhao, Y.
, 
Liu, Y.
, 
Scordato, E. S. C.
, 
Lee, M. B.
, 
Xing, X.
, 
Pan, X.
, 
Liu, Y.
, 
Safran, R. J.
, & 
Pagani‐Núñez, E.
 (2021). The impact of urbanization on body size of Barn Swallows *Hirundo rustica gutturalis*
. Ecology and Evolution, 11(1), 612–625. 10.1002/ece3.7088
33437455PMC7790637
